# Editorial: Ironome: a still untapped frontier

**DOI:** 10.3389/fmolb.2025.1651154

**Published:** 2025-07-14

**Authors:** Gloria C. Ferreira, Vladimir N. Uversky, Zoubida Karim

**Affiliations:** ^1^ Department of Molecular Medicine, Morsani College of Medicine, University of South Florida, Tampa, FL, United States; ^2^ Department of Chemistry, College of Arts and Sciences, University of South Florida, Tampa, FL, United States; ^3^ Global and Planetary Health, College of Public Health, University of South Florida, Tampa, FL, United States; ^4^ Byrd Alzheimer’s Center and Research Institute, Morsani College of Medicine, University of South Florida, Tampa, FL, United States; ^5^ INFINITY, INSERM UMR1291, CNRS UMR5051, University of Toulouse, Toulouse, France

**Keywords:** ironome, ferroptosis, iron metabolism, bacterioferritin, X-linked erythropoietic protoporphyria, leishmaniasis, Treg, intrinsically disordered protein

The ironome refers to the complete set of iron-containing proteins and iron-binding molecules across diverse organisms, starting from bacteria and unicellular parasites to complex multicellular systems such as humans. Iron is essential for life due to its redox versatility, playing vital roles in respiration, DNA synthesis, and metabolism. However, its reactivity also poses toxicity risks, necessitating precise cellular control through the ironome. Its dysregulation can trigger ferroptosis, an iron-dependent, oxidative form of regulated cell death.

Studying the ironome has revealed its central role in maintaining iron homeostasis, supporting defense against oxidative stress, and adapting to environmental challenges. Recent advances in structural biology and proteomics have expanded our understanding of diverse iron-binding architectures, including heme, iron-sulfur clusters, and ferritin-like proteins. Ongoing research on the ironome has offered new insights and perspectives into disease mechanisms, microbial pathogenesis, and therapeutic targeting of iron metabolism. Further, with the unraveling of the complexity of the ironome and the bridging of gaps between metal biochemistry and systems biology will impact health, biotechnology, and the development of antimicrobial strategies that exploit iron-dependent pathways.

Within this Research Topic we focused on recent advances in ironome research. The Research Topic collection comprises six studies or articles: Four original research articles, one review article and one mini-review article. Rajapaksha et al. reported the identification of a new protein involved in iron metabolism in the opportunistic pathogen *P. aeruginosa*. Their structural characterization reveals that the protein encoded by the *Pseudomonas aeruginosa* gene PA4880, previously annotated as a bacterioferritin, is a hybrid “Dps-like” protein (Pa DpsL). Pa DpsL exhibits the Dps (DNA-binding protein under starvation), subunit fold and bacterioferritin-type di-iron ferroxidase centers. With a 12-subunit structure, Pa DpsL catalyzes Fe^2+^ oxidation using O_2_ or H_2_O_2_, sequestering Fe^3+^ in its interior. Unexpectedly, Pa DpsL also exhibits DNA-cleaving activity, efficiently nicking and degrading plasmid DNA *in vitro*. These dual functions suggest that Pa DpsL plays a role in iron homeostasis and possibly in innate immunity. Moreover, such multifunctionality highlights novel evolutionary strategies bacteria use to manage iron while defending against genetic threats.

The ironome study by Larribau et al. identifies ferroptosis as a central mechanism of neuronal cell death in mucopolysaccharidosis type III (Sanfilippo syndrome), a lysosomal storage disorder characterized by heparan sulfate oligosaccharide (HSO) accumulation and progressive neurodegeneration. Using *in vivo* MPS III mouse models, the authors demonstrate that neuronal iron overload and lipid peroxidation drive ferroptosis, an iron-dependent, non-apoptotic cell death pathway. Notably, this process is exacerbated by impaired glutathione metabolism and dysregulated intracellular iron handling, beyond classical neuroinflammatory cascades. The neuron-specific sensitivity to ferroptosis suggests a distinct pathophysiological axis in lysosomal neurodegeneration. Therapeutic strategies aimed at modulating ferroptotic effectors, such as glutathione peroxidase 4 (GPX4), system Xc^−^, or iron chelation, could attenuate neuronal loss. These findings underscore a critical link between iron dyshomeostasis, oxidative stress, and selective neuronal vulnerability, offering a conceptual framework for targeting ferroptosis in central nervous system (CNS) disorders.

In their article, Minder et al. describe a long-term retrospective study of iron supplementation in four patients with X-linked erythropoietic protoporphyria (XLEPP), a rare disorder caused by gain-of-function mutations in the gene for erythroid 5-aminolevulinate synthase (ALAS2), leading to accumulation of protoporphyrin IX (PPIX) and zinc-protoporphyrin (ZnPP). Accumulated PPIX causes painful phototoxic reactions and risk of liver damage. The authors assessed safety, effectiveness, and treatment monitoring over a 5–8-year period. Iron supplementation, given orally or intravenously, resulted in significant reductions in erythrocyte PPIX (up to 64.5%) and ZnPP levels, as well as increases in hemoglobin and ferritin, without causing iron overload. Clinical improvements included reduced phototoxicity and normalization of liver function in one patient. Platelet counts showed an inverse correlation with PPIX levels, indicating potential usefulness as a treatment monitoring marker. While no significant adverse effects were observed, mild increased liver enzyme activities in two of the four patients suggest the need for ongoing surveillance. Minder et al. advocate standardized protocols and international data sharing to guide treatment for this rare genetic and metabolic disorder.

Uversky and Fereira dedicated their study to the analysis of the prevalence and functionality of intrinsic disorder in proteins constituting the human ironome. For the ordered ironome members, the structure-function relations are well understood, but the pervasiveness, distribution, and relevance of iron coordination and iron-binding centers in intrinsically disordered proteins remained to be evaluated. To fill this knowledge gap, this study analyzed peculiarities of intrinsic disorder in the human ironome that encompasses heme-binding proteins, proteins binding individual iron ions, and iron–sulfur cluster-binding proteins. Using a set of bioinformatic tools it was established that although the human ironome is significantly less disordered than the human calceome or the entire human proteome, many ironome members contain appreciable levels of functional intrinsic disorder. It was hypothesized that the distinct disorder predispositions observed in the ironome and calceome reflect their differing functional specializations. The lower levels of disorder in ironome can be rationalized from the major differences in how organisms handle calcium and iron, since a wide range of human diseases is associated with dysregulation of iron metabolism. These observations underscore the importance of functional intrinsic disorder in the human ironome members, where it contributes to protein-protein interactions, posttranslational modifications, and liquid-liquid phase separation.

In their review, Assouab et al. dissect the molecular strategies by which *Leishmania* parasites manipulate host ironome in cutaneous leishmaniasis (CL). Beyond expressing their own iron transporters (LIT1, LFR1), the parasites upregulate host uptake systems such as transferrin receptor 1 (TfR1) and divalent metal transporter 1 (DMT1), while downregulating NRAMP1 (SLC11A1) and inducing proteolytic cleavage of PCBP chaperones, disrupting ferritin storage, and promoting labile iron accumulation within macrophages. In parallel, infection-induced inflammatory signaling (notably IL-6) elevates hepatic hepcidin expression, triggering ferroportin degradation and iron sequestration. Unlike *Plasmodium*, which is hindered by hepcidin-driven iron restriction in hepatocytes during malaria, *Leishmania* exploits macrophage iron retention to sustain intracellular persistence, revealing a distinct evolutionary adaptation to host nutritional immunity. Thus, hepcidin modulation emerges as a strategic target for opportunistic pathogens, allowing to subvert host iron regulatory mechanisms and establish persistent infection niches.

Savagner et al. explore how ironome and mitochondrial function are coordinated to regulate CD4^+^Foxp3^+^ regulatory T cells (Tregs), a specialized T cell subset critical for maintaining immune tolerance and preventing autoimmunity. Foxp3 (Forkhead box P3) is a master transcription factor essential for the development and suppressive function of Tregs. Cellular iron homeostasis modulates TET dioxygenases involved in DNA demethylation, sustaining Foxp3 expression and epigenetic stability. Alongside, mitochondrial oxidative phosphorylation (OxPhos), driven by iron-sulfur cluster-containing enzymes, supports Treg bioenergetics. The authors discuss how mTORC1(mechanistic Target of Rapamycin Complex 1) signaling, fatty acid oxidation, and redox balance intersect to maintain Treg functionality. Under stress, dysregulated ironome promotes ferroptosis, a form of regulated cell death, threatening Treg integrity. These insights highlight iron as a master regulator of Treg identity and suggest that modulating iron and energy pathways could restore tolerance in autoimmune and inflammatory conditions.

